# Genome-Wide Classification of Myb Domain-Containing Protein Families in *Entamoeba invadens*

**DOI:** 10.3390/genes15020201

**Published:** 2024-02-02

**Authors:** Patricia Cuellar, Elizabeth J. Castañeda-Ortiz, César Rosales-Zarza, Carlos E. Martínez-Rodríguez, Israel Canela-Pérez, Mario Alberto Rodríguez, Jesús Valdés, Elisa Azuara-Liceaga

**Affiliations:** 1 Posgrado en Ciencias Genómicas, Universidad Autónoma de la Ciudad de México, Mexico City C.P. 03100, Mexico; cuellarsp2910@gmail.com (P.C.); jacqueline.castaneda.ortiz@uacm.edu.mx (E.J.C.-O.); 2 Licenciatura Ciencias Genómicas, Universidad Autónoma de la Ciudad de México, Mexico City C.P. 03100, Mexico; adrian.rosales@estudiante.uacm.edu.mx; 3 Academia de Matemáticas, Universidad Autónoma de la Ciudad de México, Mexico City C.P. 09790, Mexico; carlos.martinez@uacm.edu.mx; 4 Departamento de Bioquímica, CINVESTAV-IPN, Mexico City C.P. 07360, Mexico; israel.canela@cinvestav.mx (I.C.-P.); jvaldes@cinvestav.mx (J.V.); 5 Departamento de Infectómica y Patogénesis Molecular, CINVESTAV-IPN, Mexico City C.P. 07360, Mexico; marodri@cinvestav.mx

**Keywords:** MYB-DBD-containing proteins, encystation–excystation, transcriptional regulation, protozoan, Myb recognition element

## Abstract

*Entamoeba histolytica*, the causative agent of amebiasis, is the third leading cause of death among parasitic diseases globally. Its life cycle includes encystation, which has been mostly studied in *Entamoeba invadens*, responsible for reptilian amebiasis. However, the molecular mechanisms underlying this process are not fully understood. Therefore, we focused on the identification and characterization of Myb proteins, which regulate the expression of encystation-related genes in various protozoan parasites. Through bioinformatic analysis, we identified 48 genes in *E. invadens* encoding MYB-domain-containing proteins. These were classified into single-repeat 1R (20), 2R-MYB proteins (27), and one 4R-MYB protein. The in-silico analysis suggests that these proteins are multifunctional, participating in transcriptional regulation, chromatin remodeling, telomere maintenance, and *splicing*. Transcriptomic data analysis revealed expression signatures of *eimyb* genes, suggesting a potential orchestration in the regulation of early and late encystation–excystation genes. Furthermore, we identified probable target genes associated with reproduction, the meiotic cell cycle, ubiquitin-dependent protein catabolism, and endosomal transport. In conclusion, our findings suggest that *E. invadens* Myb proteins regulate stage-specific proteins and a wide array of cellular processes. This study provides a foundation for further exploration of the molecular mechanisms governing encystation and unveils potential targets for therapeutic intervention in amebiasis.

## 1. Introduction

*E. histolytica*, a unicellular protozoan that causes dysentery as the primary symptom of colonic invasion, is one of the most common parasitic causes of death worldwide [1]. This organism has two distinct life stages: an invasive trophozoite form and a latent cyst that is resistant to environmental changes. The process of infection starts when a person consumes contaminated food or water, then the cysts excyst in the small intestine and release the motile trophozoite [2]. Gene regulation is critical for environmental adaptation as well as for cyst conversion and pathogen transmission. Several experiments have been performed to induce encystment in *E. histolytica* where cyst-like structures have been observed; however, encystation in *E. invadens* is highly efficient and therefore remains as the model system for studying in vitro encystment development [3,4,5]. The genome of *E. invadens* is 40.88 MB long and is therefore the largest among the Entamoeba species [6,7,8]. This genome codifies 11,549 transcripts and regulates transcription through an EiCPM-GL motif (*E. invadens* core promoter motif-GAAC-like) localized 30 nt upstream from the start codon. This element resembles a fusion of the GAAC-like and Inr elements of *E. histolytica* [9]. Remarkably, no TATA box has been found, most likely due to the AT-rich nature of the genome, which makes bioinformatic searches challenging; however, a TATA-binding protein (TBP) has been identified [6,10]. Additionally, a novel transcription factor ERM-BP (Encystation Regulatory Motif-Binding Protein), a nuclear factor Y (NF-Y), and recently an EiHbox1 have been described as transcription factors involved in the encystment of these parasites [8,11,12]. Understanding the molecular mechanisms of gene expression regulation is crucial to characterize differentiation from trophozoites to cysts. Transcriptome analysis during encystation through RNAseq showed that almost 50% of all *E. invadens* genes modify their expression during this differentiation process. Besides *phospholipase D*, *Rab*, *BspA*, *phosphatases*, and genes related to cyst wall formation that overexpress during encystation, it was also observed that genes coding for MYB-DBD-containing proteins are present in this protozoan and have differential expression patterns [6]. Forty-four genes encoding for MYB-DBD-containing proteins have been previously identified in this parasite, nine containing a SHAQKYF motif, and twenty-three annotated as Myb putative or hypothetical [10,13], without any further characterization of these proteins. Changes in the expression of these proteins have been documented during cyst formation, indicating that distinct gene expression is regulated by a particular gene set at different stages of encystation. However, little is known about these transcription factors in *E. invadens*. MYB-DBD-containing proteins, from hereafter referred to as Myb proteins, have a domain related to the MYB-DBD of human c-Myb. These proteins have been described as transcription factors, coactivators, telomere-binding proteins, ribosomal binding proteins, or splicing factors [14,15,16,17,18] and belong to the homeodomain superfamily. The MYB-DBD is approximately 52 amino acid residues in length and forms a helix-turn-helix conformation with three regularly spaced tryptophan or aromatic residues with up to four imperfect conserved repeats (R) in tandem, which form a hydrophobic core [19]. Four major subfamilies of Myb proteins—1R-MYB/Myb-Related (1R), 2R-MYB (2R), 3R-MYB (3R), and 4R-MYB (4R)—are distinguished based on the number of MYB repeats in such proteins [17]. Recently, Myb proteins have been studied in unicellular organisms such as *Dictyostelium discoideum* [20,21], *Euplotes aediculatus* [22], *Tritrichomonas foetus* [23], *Trypanosoma brucei* and *Leishmania amazonensis* [24,25], *Plasmodium falciparum* [26], *Babesia bovis* [27], and *E. histolytica*, in which a wide genome analysis has been conducted [28]. In *Trichomonas vaginalis*, three different Myb proteins (TvMyb1, TvMyb2, and TvMyb3) regulate the expression of the adhesion protein AP65 [29,30]. Myb transcription factors are also important regulators of cell differentiation; for example, Myb2 in *Giardia lamblia* regulates the expression of cyst wall proteins [31,32,33], and BFD1 controls Bradyzoite differentiation in *Toxoplasma gondii* [34], as does EhMybdr in *E. histolytica* [35]. To understand the importance of Myb proteins in *E. invadens* differentiation, we performed a genomic survey of these transcription factors using c-Myb and EhMyb10 (a 2R-MYB protein of *E. histolytica*) as queries. In this study, we showed that *E. invadens* has 48 MYB-DBD-containing proteins instead of 44, as initially stated. These genes modulate their expression during encystation processes, and the proteins encoded have one, two, and four imperfect conserved repeats (R) in their MYB DNA-binding domain. Therefore, these proteins may play a crucial role as transcription modulators in *E. invadens*, enabling the invasion and formation of cysts in its reptilian host. Understanding the function and regulation of Myb proteins in *E. invadens* will allow the development of novel chemotherapeutics that could prevent cyst conversion and, consequently, disease transmission in their human counterparts.

## 2. Materials and Methods

### 2.1. Genomic Data and Identification of EiMyb-Encoding Proteins in E. invadens

Myb proteins were searched through a PSI-BLAST against the *E. invadens* IP1 genome (taxid: 33085) annotated in AmoebaDB (https://amoebadb.org/ (accessed on 29 November 2023)) [36,37] using human c-Myb (accession number P10242 UniProt database) and EhMyb10 sequences (accession number EHI_129790 from AmoebaDB) as queries. Additionally, the homeodomain term was employed in AmoebaDB and the acquired proteins were scanned in InterPro to guarantee that all Mybs were identified. The EiMyb protein sequences were retrieved from AmoebaDB and used as queries for BLASTp searches until unique MYB-DBD-containing proteins were obtained.

### 2.2. EiMyb Protein Classification

The number of repeats in the MYB-DBD (1R, 2R, 3R, or 4R) was identified using InterProScan (http://www.ebi.ac.uk/interpro/search/sequence-search (accessed on 15 November 2023)) and UniProt (https://www.uniprot.org/ (accessed on 15 November 2023)). Proteins with incomplete or distantly spaced repeats were discarded and not included in further analysis. Logos were obtained using WebLogo 3 (https://weblogo.threeplusone.com/ (accessed on 15 November 2023)) [38].

### 2.3. Multiple Sequence Alignment and Phylogenetic Analysis of EiMyb Proteins

MYB-DBD amino acid sequences were aligned using ClustalW and manually edited using Bioedit 7.0.5.3. FastTree (version 2.1.10) [39] was used to generate an approximate-maximum-likelihood phylogenetic tree based on the JTT + CAT model in VEuPathDB Galaxy workspaces. The trees were visualized with the graphical tool iTOL (version 6.8.1) [40]. OrthoMCL in AmoebaDB was used to identify proteins related by orthology or paralogy.

### 2.4. Amino Acid Sequence Analysis of EiMyb Proteins

The molecular weight (MW) and isoelectric point (p*I*) were calculated using the ProtParam tool (https://web.expasy.org/protparam/ (accessed on 15 November 2023)). Protein transmembrane helices were predicted using the TMHMM server 2.0 (http://www.cbs.dtu.dk/services/TMHMM/ (accessed on 15 November 2023)). The nuclear localization signals (NLS) were determined using http://www.moseslab.csb.utoronto.ca/NLStradamus/ and the PSORT program (https://www.psort.org/ (accessed on 15 November 2023)). Protein domain organization was performed using DOG 1.0 (https://dog.biocuckoo.org/ (accessed on 15 November 2023)) [41]. Protein structures were obtained from the alphafold.ebi.ac.uk database (last accessed: 12 May 2023), and the MYB-DBDs were visualized using the PyMOL program.

### 2.5. EiMyb Gene Expression Analysis in E. invadens

Expression patterns of *eimyb* genes were examined using the available *E. invadens* transcriptome data in AmoebaDB (https://amoebadb.org/amoeba/app/search/transcript/GenesByRNASeqEvidence (accessed on 15 November 2023)). A heat map of *eimyb* genes and transcripts per million (TPM) distribution was obtained by hierarchical cluster analysis using the pheatmap package in R software (version 3.4.3.2). Boxplot was built using the ggplot2 package in R software (version 3.4.3.2).

### 2.6. Identification of Myb Recognition Elements (MRE) in the Promoter Regions of E. invadens Genes

The presence of canonical Myb recognition element (MRE) [CT]AAC[GT]G and a C-rich sequence [CA]CCCCC, previously detected in *E. histolytica* gene promoters [28,35], was searched for using the AmoebaDB DNA motif pattern tool in the region from −500 to −1 nucleotides relative to the ATG start codon for each of the 12,007 ORFs of *E. invadens*. (Search link: https://amoebadb.org/amoeba/app/workspace/strategies/import/d20fa59ad59b609b (accessed on 15 November 2023) and https://amoebadb.org/amoeba/app/workspace/strategies/import/82af1e096fe8cfc8 (accessed on 15 November 2023)). The genes containing the identified sequences were analyzed using the STREME tool of the MEME Suite version 5.5.0 [42].

### 2.7. Analysis of Enriched Gene Ontologies

Gene ontology analysis was completed using AmoebaDB and REVIGO (http://revigo.irb.hr/ (accessed on 15 November 2023)) Version 1.8.1 [43]. The scatter plots were built using the ggplot2 package in R software (version 3.4.3.2).

## 3. Results and Discussion

### 3.1. Myb Proteins in E. invadens

To identify all ORFs that encode MYB-DBD proteins in the genome of *E. invadens*, we performed a PSI-BLAST search using the amino acid sequence of the MYB-DBDs from the human c-Myb and *E. histolytica* EhMyb10. We identified 48 genes encoding EiMyb proteins in the *E. invadens* genome; therefore, this organism possesses more proteins than its close relative, *E. histolytica*, which has 32 Myb proteins [28]. This could be because these transcription factors may regulate multiple vital functions to mediate reptilian invasion and cyst–trophozoite conversion. The 48 EiMyb proteins were retrieved from AmoebaDB and classified by the number of MYB-DBD repeats (R) using the InterPro and UniProt databases (Table 1). Forty-four of the identified EiMyb proteins match those reported by Ehrenkaufer et al. (2013) [6] and de Cadiz et al. (2013) [13] in their RNAseq analysis. Furthermore, we identified four more *eimyb* genes that were not identified in previous studies, probably because of their divergence in the MYB-DBD region. Twenty proteins were found with only one R1/R2 repeat (1R-MYB), and twenty-seven proteins had two repeats (2R-MYB). Lastly, one 4R-MYB-encoded protein was identified in *E. invadens* (Table 1), making it the first report of a four-repeat Myb protein in the Entamoeba genus.

The size of EiMyb proteins (aa), as well as computed parameters, including MW, *pI*, NLS, and subcellular localization, are listed in Table S1. EiMyb protein lengths ranged between 103 and 663 amino acids that weighed from 12.13 to 77.44 kDa with an average weight of 23.44 kDa. All proteins are defined with DNA-binding function in GO terms (Molecular Function GO: 0003677). When determining the subcellular localization, we observed that most proteins are predicted to be nuclear, and interestingly, one protein was found to be extracellular (EIN_059360). Our analysis revealed that 19 proteins have classical-type monopartite NLSs, accounting for 39.7% of proteins with 4–7 residues; 16 proteins have bipartite signals, comprising 17 amino acid residues (33.3%); and 13 proteins are NLS-free (27%). Only two proteins have a transmembrane domain (Table S1), suggesting that these proteins must be embedded in the nuclear membrane to develop their function.

### 3.2. 1R-MYB Subfamily in E. invadens

**Figure 1:**
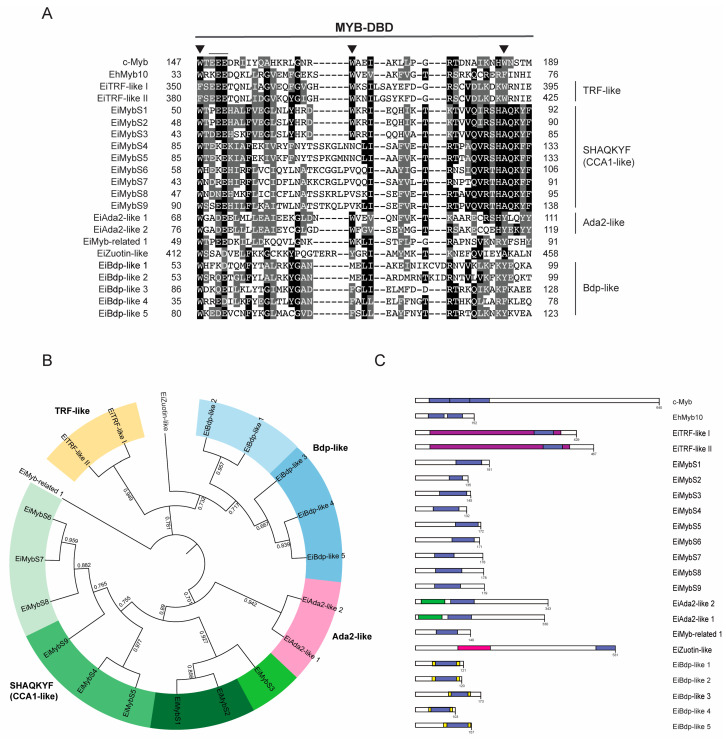
1R-MYB proteins of *E. invadens*. (**A**) ClustalW alignment of the MYB-DBD region. Arrowheads indicate conserved tryptophan residues. The acidic patch is underlined. Groups are indicated on the right. Numbers indicate the MYB-DBD position of each protein shown in the alignment. (**B**) Phylogenetic tree of the 1R-MYB proteins obtained in FastTree. The numerical values in the figure represent the reliability of the clades. The closer the value of the clade is to 1.0, the higher the confidence. Highlighted colors represent proteins belonging to the same ortholog group. EiTRF-like I (EIN_023650), EiTRF-like II (EIN_079420), EiMybS1 (EIN_086260), EiMybS2 (EIN_087120), EiMybS3 (EIN_031252), EiMybS4 (EIN_095950), EiMybS5 (EIN_224050), EiMybS6 (EIN_020720), EiMybS7 (EIN_469690), EiMybS8 (EIN_081930), EiMybS9 (EIN_407300), EiAda2-like 1 (EIN_359680), EiAda2-like 2 (EIN_390470), EiMyb-related 1 (EIN_020090), EiZuotin-like (EIN_182440), EiBdp1-like 1 (EIN_223710), EiBdp1-like 2 (EIN_034860), EiBdp1-like 3 (EIN_314460), EiBdp1-like 4 (EIN_096130), and EiBdp1-like 5 (EIN_059360). (**C**) Schematic representation of 1R-EiMyb proteins according to their size and domains. Blue, MYB-DBD; green, ADA2-lize ZZ; pink, DNAJ; purple, TRF 1 domain; and yellow, TFIIIB B”. c-Myb and EhMyb10 are used as reference.

The 1R-MYB subfamily, also referred to as Myb-related proteins, is a highly heterogeneous subfamily with several roles as TFs, chromatin remodeling proteins, and telomeric repeat-binding proteins [44,45,46]. 1R-MYBs usually contain other domains, reflecting their functional diversity. Of the 20 1R-MYB proteins in *E. invadens*, 18 were annotated as hypothetical proteins, and only 2 were annotated as putative transcriptional adapters (Table S1). The top result showed a strong resemblance to a Blast search, and the existence of the identified domains enabled us to name them EiMyb proteins (Table S2). The lengths of these 20 1R-MYB proteins ranged from 103 amino acids to 531 amino acids, with an average of 219 amino acids (Table S2). Additionally, the pI varied, ranging from 6.35 to 10.13, indicating that their functions may be distinct from one another (Table S1). The amino acid sequence alignment shows that the MYB-DBD domain is highly divergent (Figure 1A). Generally, the MYB-DBD conserves the three-spaced tryptophan residues; however, in *E. invadens*, most 1R-MYB proteins have the first tryptophan conserved, and hydrophobic amino acids substitute the second and third tryptophans (Figure 1A). The MYB-DBD is located at the N-terminal and central regions of the proteins, except for three proteins, which are located in the C-terminal region (Figure 1C). Some of these proteins harbor the SANT domain (Swi3, Ada2, human N-CoR, and the transcription factor Bdp) and are thus MYB-related [47]. SANT domains are mainly found in plants and can interact with histone tails through their acidic residues and recruit remodeling complexes [48]. Additional protein domains found in these proteins include TRFH, ADA2-like ZZ, TFIIIB B”, and the DNAJ domain (Table S2 and Figure 1C).

**Figure 2:**
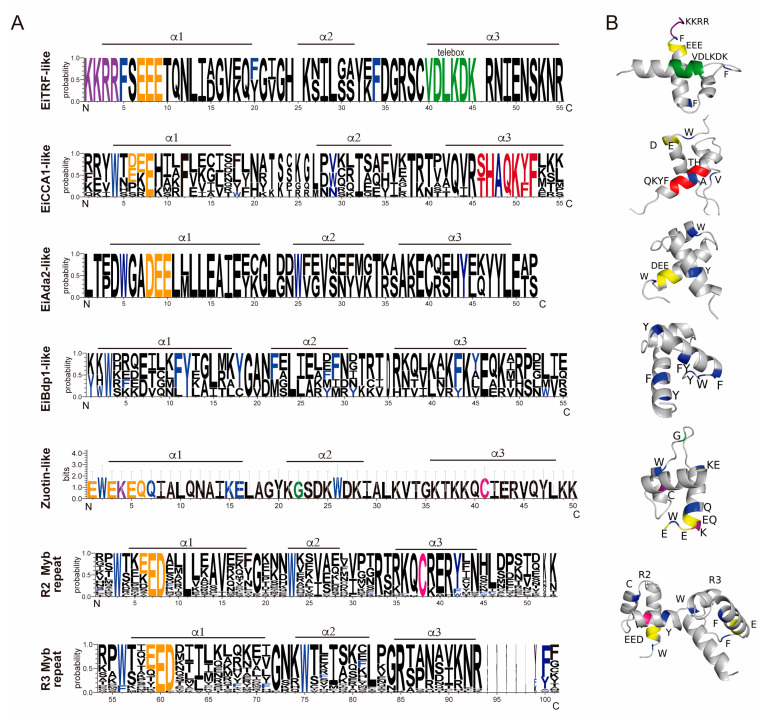
Sequence logos of the *E. invadens* Myb proteins. Multiple alignments of MYB domains were performed with ClustalW software 2.1 and visualized with WebLogo 3. (**A**) Logo of the MYB domain of EiTRF-like, EiCCA1-like, EiAda2-like, EiBdp1-like, Zuotin-like, R2 Myb repeat, and R3 Myb repeat. The *Y*-axis score indicates the probability for each position in the sequence. Black lines illustrate the position of the three α-helices in MYB-DBD. Blue: conserved hydrophobic residues; yellow: acidic patch; purple: amino-linker; green: telebox; red: SHAQKYF; and magenta: conserved cysteine of the KQCRER motif. (**B**) Molecular structures of the MYB domain of 1R and 2R-MYB proteins obtained by AlphaFold and visualized with PyMOL.

The *E. invadens* 1R-MYB proteins were then subjected to a phylogenetic study. Different clades with strong support values were identified by the tree topology*:* Zuotin, transcription factor III B’ (Bdp-like), Adaptor 2 (Ada2-like), telomeric repeat-binding factors (TRF-like), and circadian clock-associated (CCA1-like), or SHAQKYF (Figure 1B). Additionally, these proteins were also analyzed through OrthoMCL to determine the ortholog group to which they belonged. Interestingly, the same clade had different ortholog groups; for example, the CCA1-like subfamily had four different ortholog groups. This could be because, during evolution, these proteins suffered duplication events that allowed them to gain novel functions (Figure 1B). We performed multiple alignments and generated separate sequence logos for their MYB-DBD (Figure 2A). CCA1-like is the largest subgroup with nine members with the conserved SHAQK(Y/F) in the third helix of the MYB-DBD, as in *E. histolytica* proteins, and with high identity with CCA1 proteins from *Arabidopsis thaliana* [49] (Table S2). These proteins were dubbed EiMybS proteins (EiMybS1 to EiMybS9). EiMybS7 and EiMybS9 have a THAQK(Y/F) motif, where a threonine substitutes the serine (Figure 1A). The SHAQKYF-MYB proteins are common in plants, algae, and *D. discoideum*, indicating a restricted distribution in only some phyla. Studies in plants have shown that some SHAQKYF-MYBs are sequence-specific TFs that regulate the expression of clock-regulated genes and stress responses [50]. The SHAQKYF motif is localized in the third α-helix and probably because of the diversity of the CCA1-like subgroup, a second helix is not clearly observed. The CCA1-like conserves the acidic patch as well as the hydrophobic residues involved in the stability of the HTH structure (Figure 2A,B).

The TRF-like subgroup is formed by two proteins that conserve basic amino acids in the first positions (KKRR) and the telebox motif LKDKWRN (Figure 2A), which is involved in the recognition of telomeric DNA [14]. These were named EiTRF-like I and EiTRF-like II due to their high identity with TRF proteins (Table S2). The telebox motif suggests the presence of a conserved mechanism of telomeric protection in these early-branched parasites. When analyzing the molecular structure, the telebox motif conforms the first portion of the third helix that stabilizes DNA binding (Figure 2B) and therefore could be implicated in telomere recognition. This parasite possesses only two TRF-like proteins, whereas *E. histolytica* preserves three (EhTRF-like I, II, and III) [28]. This leads us to hypothesize that gene duplication occurred in *E. histolytica*.

The Ada2-like subgroup is formed by two proteins named EiAda2-like 1 and 2 that contain the ADA2-like ZZ domain. ADA2 proteins are transcriptional coactivators of the SAGA complex involved in chromatin remodeling and transcriptional regulation; they also stabilize complexes formed by direct interactions between activators and general factors in eukaryotes and were identified in *P. falciparum* [51]. Interestingly, EiAda2-like proteins may have a similar role in *E. invadens* as protein ADA2, which is a component of complexes with histone acetyltransferase. The logo sequences for the MYB-DBD region for each group (Figure 2A) show the acidic patch and the first and second conserved tryptophans that conform the clearly distinguishable HTH and a well-structured hydrophobic core (Figure 2B).

In addition, a 1R-MYB protein resembles a Zuotin protein because of the presence of a characteristic DNAJ domain and it is dubbed EiZuotin-like. Although this protein has two MYB-DBD repeats, it was classified as 1R because the second repeat is imperfect. EiZuotin-like could be related to MIDA1, a Zuotin protein in the fungus that contains two repeats of the DBD-MYB and a DNAJ domain [52]. Zuotin proteins have in vitro binding activity to tRNA and Z-DNA [53,54] and are also ribosome-associated proteins [55]. The 3-D of the DBD-MYB region of Ei-Zuotin-like shows two helix structures but a not-so-defined hydrophobic core. However, Zuotin proteins harbor, besides the DNAJ domain, an evolutionary conserved 4HB domain that serves as a linker to the SANT domain and contributes to its stability [56].

Additionally, five proteins were classified as EiBdp-like. The Bdp1 protein is one of the three subunits of the TFIIIB complex and is also termed B″. Recruitment of Pol III and promoter opening during transcription initiation depend on Bdp1. The C-terminal region of Bdp1 contains a conserved SANT domain, which normally functions as a DNA-binding module. When transcription begins, Bdp1 is situated within the Pol III active site cleft [57]. The five EiBdp-like proteins identified in *E. invadens* are significantly different from the human and *Saccharomyces cerevisiae* proteins in the sequences flanking the MYB-DBD. Four of these EiBdp-like proteins were not previously reported because of the divergence of their MYB-DBD, which can be observed in the logo generated (Figure 2A). However, the molecular structure shows two long, well-defined helixes and one short, which indicates a clear HTH structure related to a stable hydrophobic core (Figure 2B). One protein was not grouped but was considered Myb-related because of its high identity with *A. thaliana* Myb transcription factors.

When comparing the 1R-MYB subfamily, there is an evident difference between the number of Myb proteins in *E. histolytica* and *E. invadens.* A greater number of EiBdp-like proteins was observed, as three more were identified in *E. invadens* as well as a duplication of Ada proteins (two in *E. invadens* and one in *E. histolytica*). Interestingly, all nine SHAQKYF proteins are conserved in *E. histolytica* and *E. invadens,* which could suggest that these proteins play an essential role in these parasites. Finally, in contrast with the 20 1R-MYB proteins in *E. invadens*, *E. histolytica* only has 14 reported 1R-MYB proteins [28], which suggests that *E. invadens* requires a greater number of transcriptional regulators, probably because of the diversity of environments and hosts in which it develops.

In summary, all these proteins could function as transcriptional factors, telomere recognition proteins, transcription coactivators, ribosome-associated proteins, or DNAJ molecular chaperones.

### 3.3. 2R-MYB Subfamily in E. invadens

**Figure 3:**
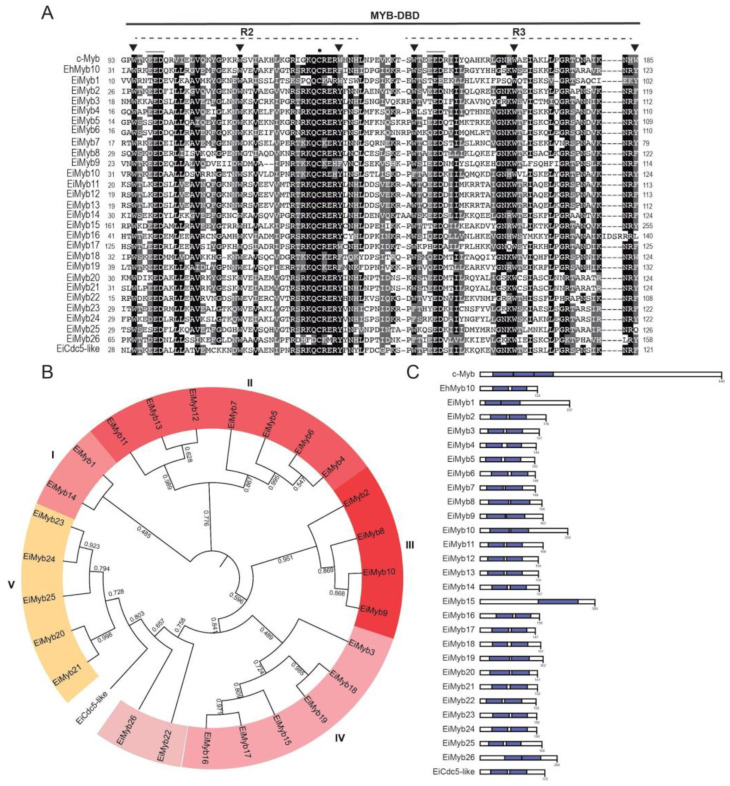
2R-MYB proteins of *E. invadens*. (**A**) ClustalW alignment of the MYB-DBD region. Arrowheads indicate conserved tryptophans. Numbers indicate the MYB-DBD position of each protein shown in the alignment. The acidic patch is underlined, and R2 and R3 repeats are indicated as dotted lines. The black circle indicates the conserved cysteine residue in the R2 repeat. (**B**) Phylogenetic tree of the 1R-MYB proteins obtained in FastTree. The numerical values in the figure represent the reliability of the clades. The closer the value of the clade is to 1.0, the higher the confidence. Highlighted colors represent proteins belonging to the same ortholog group. EiMyb1 (EIN_284910), EiMyb2 (EIN_178740), EiMyb3 (EIN_047330), EiMyb4 (EIN_206260), EiMyb5 (EIN_169560), EiMyb6 (EIN_168610), EiMyb7 (EIN_207200), EiMyb8 (EIN_022390), EiMyb9 (EIN_080130), EiMyb10 (EIN_276810), EiMyb11 (EIN_307410), EiMyb12 (EIN_308550), EiMyb13 (EIN_308550), EiMyb14 (EIN_095310), EiMyb15 (EIN_399710), EiMyb16 (EIN_490880), EiMyb17 (EIN_310240), EiMyb18 (EIN_425382), EiMyb19 (EIN_046410), EiMyb20 (EIN_183110), EiMyb21 (EIN_183730), EiMyb22 (EIN_169190), EiMyb23 (EIN_359630), EiMyb24 (EIN_379820), EiMyb25 (EIN_168860), EiMyb26 (EIN_405040), and EiCdc5-like (EIN_248780). (**C**) Schematic representation of Myb proteins according to their size and domains. Blue, MYB-DBD. c-Myb and EhMyb10 are used as references.

The most prevalent subfamily in *E. invadens*, as well as in *E. histolytica*, is the 2R-MYB subfamily, also known as R2R3-Myb proteins. This 2R-MYB subfamily comprises 27 ORFs that were named numerically; interestingly, they have more similarity to plant 2R-MYB proteins than to *Homo sapiens* c-Myb (Table S2). These proteins are mostly annotated as transcription factors related to *A. thaliana* Myb proteins (transcription factor MYB, putative; transcription factor WEREWOLF, putative; trichome differentiation protein GL1, putative; r2r3-MYB transcription factor, putative, or C-MYB, putative) in AmoebaDB, although there are three proteins annotated as hypothetical. 2R-MYB are the most abundant Myb proteins in plants and are involved in a variety of biological activities, including seed development, morphogenesis, meristem formation, secondary cell wall production, and hormonal signal transmission [58,59]. Although these proteins greatly conserve their MYB-DBD, their N- and C-termini are divergent, often having residues in disordered regions that may undergo post-translational modifications and therefore could affect transcription factor stability or localization [60]. These proteins contain two repeats in their MYB-DBD. The size of these proteins ranges from 145 to 305 amino acids and is similar to that of their *E. histolytica* counterparts, with a molecular weight of 17.21 kDa to 36.47 kDa, respectively. The *pI* of R2R3-Myb proteins varied from 6.18 to 10.08 (Table S1). These proteins are predicted to be localized in the nucleus, and in some cases, a nuclear localization signal was predicted, supporting their role in transcriptional regulation (Table S1). These proteins contain two repeats in their MYB-DBD. Alignment analysis of the MYB-DBD revealed that the first and second tryptophan residues of repeats 2 and 3 are conserved; nevertheless, substitutions with aromatic residues are present in the third tryptophan of both repeats, often replaced by tyrosine or phenylalanine residues (Figure 3A).

The highly conserved patch of acidic residues, such as glutamic or aspartic acid, is common to all Myb-related domains and is also in 2R-MYB proteins in *E. invadens* (Figure 3A). These acidic residues are positioned in the first of the α-helices within each of the two repeats that comprise the MYB-DBD (Figure 3B). In c-Myb, the acidic residues are relevant for transcriptional activity, chromatin binding, and interaction with the H4 histone N-terminal tail [61]. A conserved cysteine residue in the third helix of the R2 domain of all the *E. invadens* 2R-MYB proteins was also present, forming the QCRER motif (Figure 3A), as in the *E. histolytica* R2R3Myb proteins. This motif can be observed in the third helix of the R2 repeat near the acidic residues localized in the first helix (Figure 2B). The conserved cysteine is relevant for REDOX-dependent DNA binding in mammals, plants, and other eukaryotic organisms [62]. Next, we performed a phylogenetic analysis of the 2R-MYB proteins (Figure 3B), which were further divided into five subgroups (I, II, III, IV, and V), except for three protein sequences that could not be grouped. Altogether, these proteins belong to three different ortholog groups determined by OrthoMCL-DB (Figure 3, Table S2). In most cases, the MYB-DBD is located in the middle of the polypeptide and comprises almost the total length of the protein (Figure 3C). Finally, the protein with accession number EIN_248780 presents a high identity with the CDC5 protein from *H. sapiens* and *A. thaliana* and was therefore dubbed EiCDC5-like. CDC5 proteins have two MYB repeats followed by a third imperfect MYB-like repeat, or D3 domain. In *S. cerevisiae*, the ortholog of CDC5 has been reported to play a role in pre-mRNA splicing [16], but it also functions as a transcription factor in plants that recognize the DNA-binding consensus CTCAGCG, showing multiple roles in transcriptional regulation [63].

### 3.4. 4R MYB-DBD Protein

**Figure 4:**
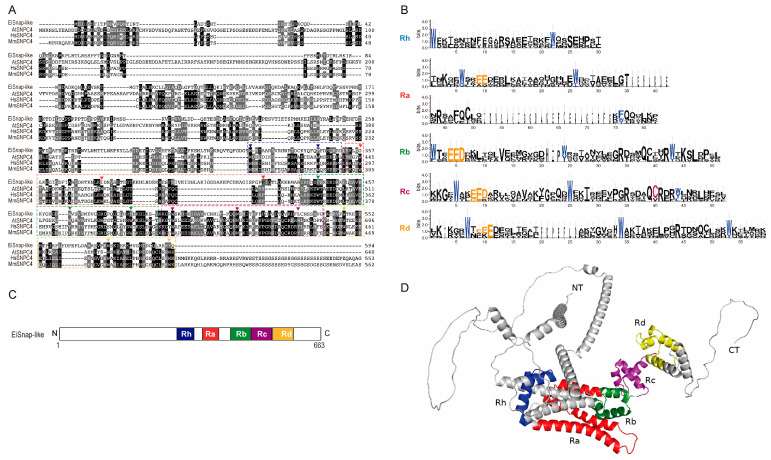
4R-MYB protein of *E. invadens*. (**A**) ClustalW alignment of the amino-terminal region of EiSnap-like and SNAPc orthologs from *A. thaliana*, *H. sapiens*, and *Mus musculus*. Arrowheads indicate the conserved tryptophans, and the dotted line indicates the four adjacent MYB repeats, Ra, Rb, Rc, and Rd (red, green, brown, and yellow dotted boxes), with an additional half MYB repeat (Rh) in front of Ra (blue dotted box). (**B**) Sequence logos generated from the multiple sequence alignment of the analyzed ortholog 4R-MYB proteins. (**C**) Schematic diagram of EiSnap-like visualized with Dog 2.0 (**D**) Three-dimensional structure of EiSnap-like protein performed in AlphaFold and visualized with PyMOL.

With 663 amino acids, EIN_267690 encodes the largest Myb protein found in *E. invadens,* and interestingly, it has no detectable nuclear localization signals (Table S1). In AmoebaDB, this protein is annotated as snap190 putative, with 26.47% identity to c-Myb and 22.9% and 23.03% identity to SNPC4 from *H. sapiens* and *A. thaliana*, respectively (Table S2). The MYB-DBD from EiSnap-like exhibits substantial conservation of amino acid residues, which are essential for the sequence-specific binding of the promoter region of snRNA genes [63,64] (Figure 4A,B). The EiSnap-like MYB-DBD has four MYB repeats: Ra, Rb, Rc, and Rd, and an additional half MYB repeat (Rh) situated N-terminal to Ra according to the nomenclature used for the HsSNAPc4 (Figure 4C) [64]. The 3-D structure shows a mostly helicoidal conformation and non-structured NT and CT regions that could contribute to regulating EiSnapAP-like (Figure 4D). 4R-MYB has been reported as the small nuclear RNA (snRNA)-activating protein complex subunit that participates in the transcription initiation of snRNAs in plants [65]. Both RNA polymerase II and III snRNA gene transcription require the complex SNAPc, in which SNAP190 proteins participate. Most eukaryotes have SNAPc, which can have three or five subunits depending on the species [64,66]. Interestingly, the SNAP proteins have been identified in the Excavata group, including *G. lamblia*, *Leishmania major*, *T. brucei*, and *Naegleria gruberi*, with 64% identity [67]. As was mentioned earlier, no 4R-MYB proteins have been previously discovered in *E. histolytica*; however, the protein encoded by the locus EHI_130710 is considered its ortholog in the AmoebaDB database. Therefore, it would be interesting to identify if it indeed possesses a 4R-MYB as well as the genes that are regulated by these proteins in both parasites.

### 3.5. Expression Analysis of the EiMyb Genes during Trophozoite Differentiation

**Figure 5:**
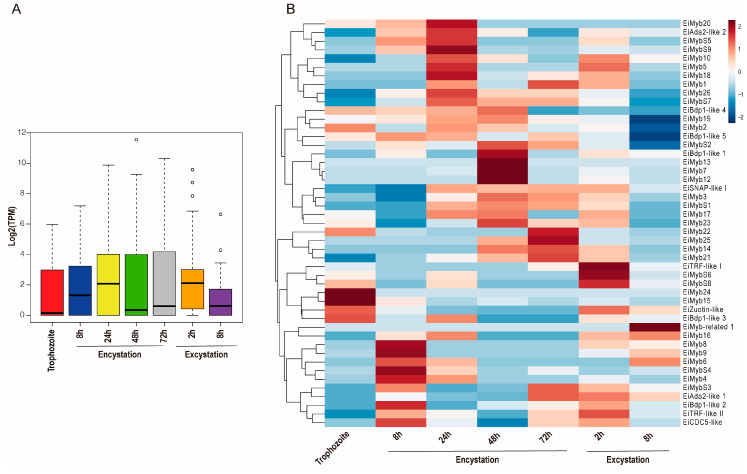
Expression profile of *E. invadens* Myb genes during encystation and excystation. (**A**) Boxplot showing the number of *eimyb* genes expressed in each condition analyzed during trophozoite–cyst differentiation. The middle lines in the boxplot represent the median, and circles represent outliers. (**B**) Hierarchical clustering heatmap of *eimyb* genes; each column represents a gene and each row represents a condition. The colors in the graph represent the sample’s level of gene expression [Log2 (TPM)]. Blue signifies that the gene expression is low in the sample, whereas red shows that the gene is strongly expressed. Data were obtained from AmoebaDB.

Focused on cyst differentiation, the transcriptome dataset obtained through RNAseq data from AmoebaDB was analyzed. We analyzed the expression profiles of *eimyb* genes from encysting (8, 24, 48, and 72 h after transfer to encystation media) and from excysting parasites (2 and 8 h after induction of excystation) [6]. When analyzing the median and distribution of expression values of all *eimyb* genes in trophozoite differentiation, we observed an upregulation during late encystation (24–72 h) (Figure 5A). The expression patterns of the 48 *eimyb* genes in *E. invadens* under encysting conditions were visualized using a heatmap analysis. We observed that only 14 were expressed in the trophozoite stage, with *eimyb15* and *eimyb24* having the greatest expression (Figure 5B). In addition, *eimyb24* is a trophozoite-specific gene. In *E. histolytica*, its ortholog is EhMyb10, which means that EhMyb10 could be essential for the parasite and therefore a potential target for therapy. Twenty *eimyb* genes modulate their expression during cyst differentiation; therefore, we searched for signatures that could suggest stage-specific Myb proteins (Figure 5B). During early encystation (8h), 23 *eimyb* genes are expressed, with *eimyb9* and *eimybs4* being the most expressed. During encystation progression (24, 48, and 72 h), 23, 30, and 23 *eimyb* genes are expressed (Figure 5B). At 24 h, *eimybs9*, *eimyb18,* and *eimyb20* have the greatest expression. In late encystation (48 h), *eimyb7*, *eimyb12*, and *eimyb13* are most expressed. Interestingly, these three genes appear as a specific signature for this encystment time (Figure 5B). At 72 h of encystation, *eimyb22* and *eimyb25* express the most. On the other hand, excystation is an important process that ensures *E. invadens* dissemination; interestingly, during early excystation times (2 h), the greatest number of *eimyb* genes is expressed (35 *eimyb* genes).

This could be due to the parasite’s need to reactivate transcription and initiate reptilian host invasion, as previous studies have shown that from the total transcriptome, 1025 and 1032 genes are upregulated at 2 h and 8 h, respectively [13]. At 2 h of excystation, *eimybs6*, *eimybs8*, and *eitrf-like 1* show the greatest expression. At 8 h of excystment, only 14 *eimyb* genes are expressed, and *eimyb-related 1* has the greatest expression and is specific to this stage time (Figure 5B). Altogether, these data suggest that while widely expressed *eimybs* may control the transcription of a large number of genes, a specific set of EiMyb proteins is required to modulate the spatial–temporal expression patterns during trophozoite–cyst differentiation. Therefore, it is important to study the genes that are regulated through this Myb-selective expression. In agreement, we did not observe a constitutive expression pattern of any of the *eimyb* genes, which reinforces their specific role during parasite development. This could explain why, in other studies, only a subset of cyst-specific genes is induced when a single *eimyb* gene is overexpressed [35]. Interestingly, the gene that codes for EiCDC5-like, a protein similar to CDC5 that participates in splicing, suggests that splicing might be a necessary process in early encystation–excystation (8 h encystation). This is interesting because almost 26% of the expressed genes contain introns (1536 from 5894 genes with introns from genome annotation) [6], suggesting the necessary participation of the spliceosome in these stages. Lastly, *eitrf-like I* and *II* are expressed in specific stages (Figure 5B) in which replication occurs, as nuclear division is necessary for encystment; therefore, these proteins could be required for telomeric protection. TRF-like proteins have been identified and characterized in *T. brucei*, *T. cruzi*, *L. major,* and *E. histolytica*, where their role as telomere DNA-binding proteins has been reported to provide a possible function in telomere-end protection [24,25,68].

### 3.6. Presence of the Myb Recognition Element (MRE) and the C-Rich Sequence in E. invadens Gene Promoters

To identify the target genes of EiMyb proteins, we searched for in silico Myb recognition elements in *E. invadens* gene promoters through two DNA sequences previously identified in *E. histolytica*: the canonical Myb recognition element (MRE) and a C-rich sequence [28,35]. In this analysis, 2559 genes had the canonical MRE in their putative regulatory region (−500 to −1 pb from ATG); 1700 genes were annotated as hypothetical; and 859 had predicted functions. In contrast, 288 genes had a C-rich sequence in the promoter region (192 hypothetical and 96 with predicted functions). The MRE and C-rich sequences were confirmed through STREME (Table 2). Interestingly, the signatures of both sequences had slight differences depending on the encystation or excystation stage (Table 2). Subsequently, we observed that 815 and 838 genes modified at least twofold their expression during encystment and excystment, while 99 and 100 genes modified their expression during encystment and excystment, respectively, for MRE and C-rich sequences (Table 2). As for their localization, these sequences are distributed throughout the promoter, in contrast with the reported EiCPM-GL motif, which is clearly positioned in the first 50 nucleotides of this parasite in about 15% of these genes [9]. Further experimental analysis could confirm that these signatures are recognized by EiMyb proteins.

### 3.7. Functions of the Putative EiMyb Target Genes

**Figure 6:**
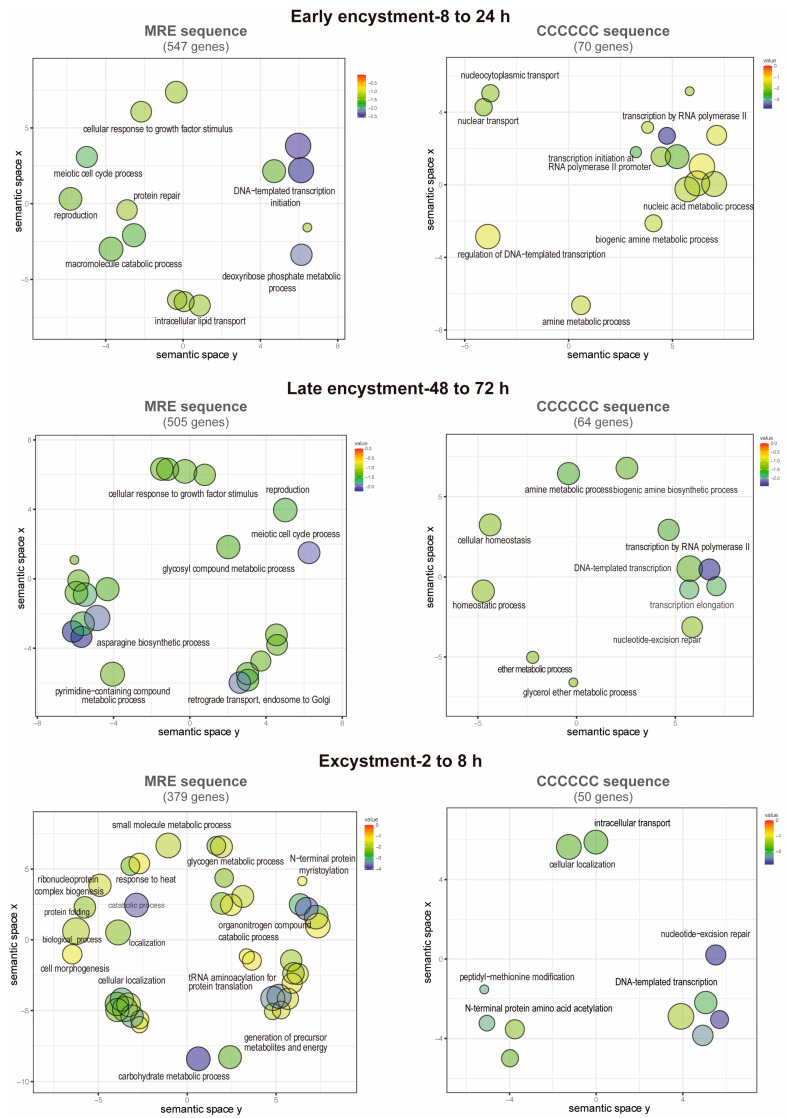
GO annotations of upregulated genes during cyst differentiation in *E. invadens*. Biological process annotations associated with genes containing the MRE and the C-element in their promoters are visualized using a two-dimensional semantic space scatterplot. The spatial organization is based on semantic similarity. The number of node labels is minimized to allow visualization of the node colors on the scatterplot. The score equals the *p*-value for each GO annotation term node. Blue nodes indicate more significant *p*-values and red nodes indicate less significant *p*-values.

**Figure 7:**
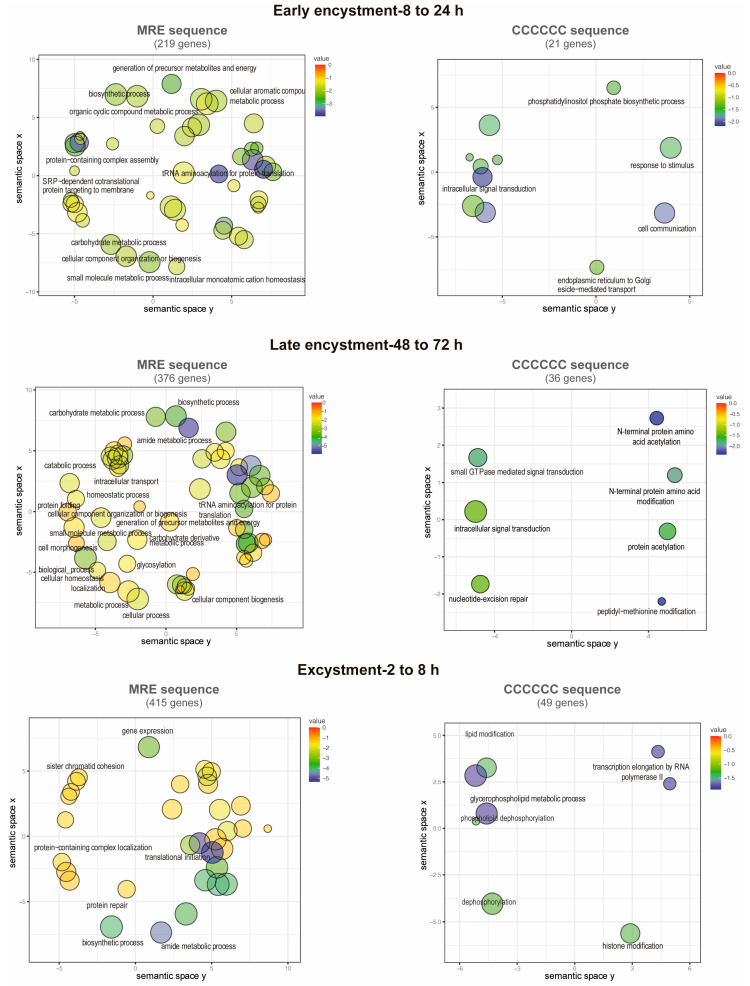
GO annotations of downregulated genes during cyst differentiation in *E. invadens*. Biological process annotations associated with genes containing the MRE and the C-element in their promoters are visualized using a two-dimensional semantic space scatterplot. The spatial organization is based on semantic similarity. The number of node labels is minimized to allow visualization of the node colors on the scatterplot. The score equals the p-value for each GO annotation term node. Blue nodes indicate more significant *p*-values and red nodes indicate less significant *p*-values.

Term enrichment analysis was performed on the previous genes to identify GO categories related to biological processes. Notably, 547 MRE-containing genes upregulate during early encystment and are related to catabolism because cellular metabolism decreases in this stage (Figure 6). An interesting finding was that in early and late encystment (505 total upregulated genes), many DNA and RNA processing genes are upregulated, probably to prepare the cell for encystment and regulate its gene expression. Nuclear division is an important event during encystment to generate tetra-nucleated cysts, and for this, DNA replication must be present, which is represented by meiotic nuclear division and DNA repair-upregulated genes. In *E. invadens*, encystation is accomplished by multinucleation events that could benefit the parasite by allowing genomic changes and recombination [69]. In agreement, during encystation, it has been observed that meiotic-related genes are expressed [6]. We also found genes related to the secretion process and exocytosis that could be related to the transportation of cyst-forming components to the cell membrane (Figure 6) [70]. During excystment, 379 genes were upregulated, and an increase in metabolism-related genes was observed, as well as genes related to temperature stimulus responses and the reproductive process. The sexual pathway is induced by the stress response to starvation, as in many eukaryotes such as yeast and *Dictyostelium* [69]. This finding is in agreement with the overexpression of meiotic and homologous recombination genes reported by Ehrenkaufer et al. (2013) [6] during stage conversion. Furthermore, downregulated genes are represented by a metabolic process decrease related to glucose and energy uptake as well as organelle and protein biogenesis (219, 376, and 415 downregulated genes in early and late encystment and excystment, respectively) (Figure 7).

On the other hand, the gene ontology terms associated with the C-rich sequence showed genes involved in cyst formation and were also enriched in genes involved in post-translational modifications (70 and 64 genes upregulated in early and late encystment, respectively). During excystment, among the 47 upregulated genes, we found some related to transcription initiation, probably reactivating the transcription of many genes during this process. Finally, the downregulated genes (21, 36, and 49 genes in early and late encystment and excystment, respectively) were mostly related to intracellular signal transduction and transcription initiation. We suggest that MYB-DBD proteins could recognize both an MRE element and a C-rich sequence to regulate gene expression in *E. invadens*; however, its genome is approximately 70% AT-rich, which could be related to an increase in the number of MRE identified (2559 vs. 288 genes).

Finally, in *E. histolytica*, the expression of Myb transcription factors in trophozoite is related to invasive amoeba [71]. It may be that EiMyb proteins are also related to the ability to infect and invade all tissues of reptilian hosts. On the other hand, encystation in *E. invadens* is triggered by glucose starvation, which in *E. histolytica* is related to the overexpression of some Myb proteins. It is important to mention that 41.95% of the *E. invadens* proteome is common among other species, and the difference in the proteome could be related to the ability to infect different species of reptiles. Therefore, it is understandable that the parasite needs a greater family of transcription factors to respond selectively to the host.

## 4. Conclusions

In this study, we searched for *E. invadens* MYB-domain-containing proteins, and 48 genes encoding for these proteins were identified and classified, as well as thoroughly described in this work. Most EiMyb proteins have domains that are involved in transcription initiation, such as ADA-2, SWI complex I, and Reb1, among others. Expression analysis during encystation–excystation obtained from the AmoebaDB database showed that genes encoding MYB-domain-containing proteins were differentially expressed, some of them only in the trophozoite stage, others mainly in the cyst stage. This indicates that EiMyb proteins may regulate the expression of stage-specific proteins and a great variety of cellular processes in this parasite. The elucidation of the function and regulation of EiMyb proteins in the *E. invadens* stage transition may lead to the discovery of targets for the development of new chemotherapeutics that interfere with cyst conversion. Also, knowing how Myb proteins tune cyst conversion could help elucidate how the process is executed in *E. histolytica* and promote encystation in vitro through Myb overexpression or repression.

## Figures and Tables

**Table 1:** MYB-DBD-containing proteins in *E. invadens* retrieved from AmoebaDB and classified according to their number of DBD-MYB repeats and motifs.

Myb Subfamily	Groups	Number of Members
1R-MYB	SHAQKYF (CCA1-like)	9
	Bdp1-like	5
	TRF-like	2
	Ada2-like (Transcriptional adapter putative-Ada2)	2
	Myb-related	1
	Zuotin-like	1
2R-MYB	Myb transcription factors	13
	Trichome differentiation protein GL1 related	6
	WEREWOLF transcription factors related	3
	Hypothetical proteins	3
	R2R3-Myb transcription factors	2
4R-MYB	Snap190	1
Total		48

**Table 2:** MRE and CCCCCC motif search in *E. invadens* gene promoters (−500 to −1 pb from ATG).

Motif Consensus Sequence	Modified Sequence	Motif Containing Genes *	E-Value ^a^	STREME Confirmed	Stage-Related Genes
[T/C]AAC[G/T]G	CAACTG	2559 (21.31%)	2.0 × 10^−36^	2541 (99.3%)	Trophozoite
	YAACTG	815 (6.78%)	8.0 × 10^−13^	810 (99.3%)	Encystation
	CAACTG	838 (6.97%)	5.3 × 10^−17^	834 (99.5%)	Excystation
[CA]CCCCC	MCCCCC	288 (2.39%)	6.2 × 10^−6^	284 (98.6%)	Trophozoite
	CCCCCC	99 (0.82%)	2.1 × 10^−1^	97 (98.0%)	Encystation
	CCCCCC	100 (0.83%)	9.8 × 10^−2^	98 (98.0%)	Excystation

* Search performed against 12,007 ORFs identified in AmoebaDB. Y: C or T; M: A or C. ^a^ The E-value is the *p*-value (*p* < 0.05) multiplied by the number of motifs reported by STREME.

## Data Availability

The data presented in this study are available on request from the corresponding author. The data are not publicly available due to privacy.

## Supplementary Materials

The following supporting information can be downloaded at https://www.mdpi.com/article/10.3390/genes15020201/s1, Table S1: Characteristics of all the full length EiMyb proteins of *E. invadens* classified according to their repeat number. Table S2: EiMyb proteins of *E. invadens* named and classified according to their homology to *H. sapiens* and *A. thaliana*.
