# Supplementary material for: Genome-Wide Classification of Myb Domain-Containing Protein Families in Entamoeba invadens
Source: Genes (Basel). 2024 Feb 2;15(2):201. doi: 10.3390/genes15020201 (PMC10887745; doi:10.3390/genes15020201)
Supplement: Supplementary file 1 [file genes-15-00201-s001.zip › Table S1.pdf]

Supplementary Table S1. Characteristics of all the full length EiMyb proteins of *Entamoeba invadens* classified according to their repeat number

	No	Gene ID	Annotation	Size (aa)	MW (kDa)	pI	TH [a]	Subcellular localization [b]	NLS1 sequence	NLS1 Description	NLS2 sequence	NLS2 Description
1R-Myb	1	EIN_020090	Hypothetical protein	140	16.37	9.45	-	N	KKRK	Monopartite of 4 residues		
	2	EIN_020720	Hypothetical protein	145	17.01	9.42	-	C, N	KKKK	Monopartite of 4 residues		
	3	EIN_023650	Hypothetical protein	429	49.25	7.52	Y	C, N	KKKKRR	Monopartite of 6 residues		
	4	EIN_031250	Hypothetical protein	177	20.26	9.88	-	N	KKVRKQYTLTXRRR	Monopartite of 14 residues		
	5	EIN_034860	Hypothetical protein	120	14.39	9.51	-	C, N	KKPKVQQRRKEDLKPR K	Bipartite of 17 residues		
	6	EIN_059360	Hypothetical protein	157	18.17	8.87	-	C, E	-			
	7	EIN_079420	Hypothetical protein	467	53.12	8.4	Y	N	PKKAKTT	Monopartite of 7 residues	KKKKRR	Monopartite of 6 residues
	8	EIN_081930	Hypothetical protein	135	15.75	9.51	-	C, N	-			
	9	EIN_086260	Hypothetical protein	179	20.87	10.13	-	N	RKQRKQYTITXXKKREV	Bipartite of 17 residues		
	10	EIN_087120	Hypothetical protein	178	20.64	9.69	-	N	RKQRKQYTITXXKKREV	Bipartite of 17 residues		
	11	EIN_095950	Hypothetical protein	172	20.19	9.19	-	C, N	-			
	12	EIN_096130	Hypothetical protein	103	12.13	9.27	-	C, N	-			
	13	EIN_182440	Hypothetical protein	531	62.75	8.63	-	C, N	RRWMNKENEKKTTKLR K	Bipartite of 17 residues	KRIWRLAELAXHKKDPR	Bipartite of 17 residues
	14	EIN_223710	Hypothetical protein	121	14.47	9.42	-	C, N	RKRHK	Monopartite of 5 residues		
	15	EIN_224050	Hypothetical protein	171	20.08	9.71	-	C, N	-			
	16	EIN_314460	Hypothetical protein	173	20.08	8.94	-	C, N	KRVVAKPRKSXXKKWD K	Bipartite of 17 residues		
	17	EIN_359680	transcriptional adapter, putative	330	38.84	6.45	-	C, N	KRLEQYYKMYIERIRVR	Bipartite of 17 residues	RREGCLTMEEXTKERRR	Bipartite of 17 residues
	18	EIN_390470	transcriptional adapter, putative	343	40.46	7.55	-	C, N	RKLERYTKMCLERKRIR	Bipartite of 17 residues	RKGAKGAKAAXHKGGK K	Bipartite of 17 residues
	19	EIN_407300	Hypothetical protein	181	20.76	6.35	-	C, N	-			
	20	EIN_469690	Hypothetical protein	132	15.58	9.61	-	C, N	PEIRRKV	Monopartite of 7 residues		
2R-Myb	21	EIN_022390	transcription factor MYB90, putative	164	18.72	9.53	-	N	KRSQNNKENAXXLKKK L	Bipartite of 17 residues		
	22	EIN_046410	MYB, putative	167	19.62	9.67	-	N	-			
	23	EIN_047330	transcription factor WEREWOLF, putative	157	18.22	9.8	-	N	KHKK	Monopartite of 4 residues	PGRTRKQ	Monopartite of 7 residues
	24	EIN_080130	trichome differentiation protein GL1, putative	167	19.42	8.77	-	N	PNRTKKQ	Monopartite of 7 residues		
	25	EIN_095310	transcription factor MYB75, putative	157	17.97	9.8	-	N	PGRTRKQ	Monopartite of 7 residues		
	26	EIN_168610	trichome differentiation protein GL1, putative	148	17.49	9.58	-	N	KKIEIFVVGRXNRKQCR	Bipartite of 17 residues		
	27	EIN_168860	transcription factor MYB59, putative	164	19.5	9.32	-	N	RKKRK	Monopartite of 5 residues		

	28	EIN_169190	C-MYB, putative	150	17.64	9.77	-	N	-			
	29	EIN_169560	r2r3-MYB transcription factor, putative	145	17.21	9.79	-	N	KKVEKNIKGRXNRKQC R	Bipartite of 17 residues	KRPWTIEEDQTIMKTRK	Bipartite of 17 residues
	30	EIN_178740	transcription factor WEREWOLF, putative	176	20.68	9.78	-	N	KRANSKTKEVXXKQKE K	Bipartite of 17 residues		
	31	EIN_183110	transcription factor MYB23, putative	151	17.41	9.75	-	N	HKKK	Monopartite of 4 residues		
	32	EIN_183730	Hypothetical protein	152	17.52	9.37	-	N	PVSKRPR	Monopartite of 7 residues		
	33	EIN_206260	trichome differentiation protein GL1, putative	149	17.42	9.47	-	N	KKVELFVPKRXNRKQCR	Bipartite of 17 residues		
	34	EIN_207200	transcription factor MYB23, putative	148	17.55	9.11	-	N	KHKK	Monopartite of 4 residues	PERTKKQ	Monopartite of 7 residues
	35	EIN_248780	transcription factor MYB23, putative	237	27.3	9.52	-	N	-			
	36	EIN_276810	Hypothetical protein	233	27.58	9.87	-	N	PNRTKKQ	Monopartite of 7 residues		
	37	EIN_284910	transcription factor WEREWOLF, putative	172	19.87	9.73	-	N	KKKK	Monopartite of 4 residues	PNRSRKQ	Monopartite of 7 residues
	38	EIN_307180	transcription factor MYB48, putative	155	18.55	9.77	-	N	KRAWTQEEDDXIIMKRR	Bipartite of 17 residues		
	39	EIN_307410	transcription factor MYB90, putative	168	19.84	9.77	-	N	KRAWTQEEDDXIIMKRR	Bipartite of 17 residues		
	40	EIN_308550	transcription factor MYB75, putative	155	18.46	9.68	-	N	KRAWTQEEDDXIIMKRR	Bipartite of 17 residues		
	41	EIN_310240	transcription factor MYB23, putative	147	17.13	9.72	-	N	PFKKKYS	Monopartite of 7 residues	PSRTRKQ	Monopartite of 7 residues
	42	EIN_359630	trichome differentiation protein GL1	150	17.29	9.68	-	N	-			
	43	EIN_379820	trichome differentiation protein GL1, putative	150	17.32	10.08	-	N	-			
	44	EIN_399710	Hypothetical protein	305	36.47	9.88	-	N	KKGAHLLIIFQSKKKVK	Bipartite of 17 residues	RRWHLVALKIXPSRTRK	Bipartite of 17 residues
	45	EIN_405040	trichome differentiation protein GL1, putative	204	23.3	6.18	-	N	KRKK	Monopartite of 4 residues		
	46	EIN_425380	transcription factor MYB48, putative	161	18.6	9.34	-	N	-			
	47	EIN_490880	r2r3-MYB transcription factor, putative	158	18.42	9.46	-	N	KKKK	Monopartite of 4 residues	PGRSRKQ	Monopartite of 7 residues
4R-Myb	48	EIN_267690	snap190, putative	663	77.44	7.47	-	C, N	-			

[a] -, non detected; y, yes

[b] C, cytoplasm; n, nucleus; E, extracellular
