# Supplementary material for: Genome-Wide Classification of Myb Domain-Containing Protein Families in Entamoeba invadens
Source: Genes (Basel). 2024 Feb 2;15(2):201. doi: 10.3390/genes15020201 (PMC10887745; doi:10.3390/genes15020201)
Supplement: Supplementary file 1 [file genes-15-00201-s001.zip › Table S2.pdf]

Supplementary Table S2. EiMyb proteins of *E. invadens* named and classified according to their homology to *H. sapiens* and *A. thaliana*

Group	Gene ID	Gene (pb)	mRNA	Protein name	DBD-MYB	InterProScan domains	CD-Search domains	*H. sapiens* c-Myb	*A. thaliana* [a]	*E. histolytica*	OrthoMCL[b]
**1R-MYBS**											
**TRF-like**	EIN_023650	1290	1290	EiTRF-like I	348-392	Telomeric Repeat Binding Factor 1// TM Helix	SANT_TRF/SANT Superfamily	29.07% 7e-12 29.90% 3e-17 (TERF1)	48.08% 3e-13 (CAD531509.1)	49.89% 5e-133 (EHI_148140)	OG6_105798
	EIN_079420	1404	1404	EiTRF-like II	378-422	Telomeric Repeat Binding Factor 1// TM Helix	SANT_TRF/SANT Superfamily	26.32% 4e-14 35.87 8e-17 (TERF1)	21.54% 2e-15 (OAP03200.1)	55.13% 5e-164 (EHI_001110)	OG6_105798
**CCA-like (SHAQKYF)**	EIN_086260	540	540	EiMybS1	48-92	Myb-DNA Binding/SANT Superfamily	Myb-DNA Binding/SANT Superfamily/RSC8 Chromatin remodeling	22.95% 4e-14	64.71% 6e-25 (AAF23291.1)	63.10% 2e-45 (EHI_092160)	OG6_204742
	EIN_087120	537	537	EiMybS2	46-90	Myb-DNA Binding/SANT Superfamily	Myb-DNA Binding/SANT Superfamily	21.92% 1e-14	45.37% 6e-22 (AAF81310.1)	52.43% 2e-35 (EHI_092160)	OG6_204742
	EIN_031250*	601	534	EiMybS3	41-85	Myb-DNA Binding/SANT Superfamily	Myb-DNA Binding/SANT Superfamily	25.45% 2e-14	58.06% 6e-20 (OAP07468.1)	54.55% 4e-35 (EHI_136420)	OG6_167769
	EIN_095950	519	519	EiMybS4	83-133	Myb-DNA Binding/SANT Superfamily	SANT Superfamily	16.13% 1e-11	20.83% 3e-12 (CAA0383923.1)	38.22% 1e-18 (EHI_155580)	OG6_127050
	EIN_224050	516	516	EiMybS5	83-133	Myb-DNA Binding/SANT Superfamily	SANT Superfamily	25.64% 2e-09	27.78% 7e-12 (NP_00107786.1)	39.10% 6e-17 (EHI_155580)	OG6_127050

	EIN_020720	438	438	EiMybS6	56-106	Myb-DNA Binding/SANT Superfamily	SANT Superfamily	-	24.29% 2e-12 (CAA0367555.1)	62.02% 5e-36 (EHI_051440)	OG6_204588
	EIN_469690	399	399	EiMybS7	41-91	Myb-DNA Binding/SANT Superfamily	SANT Superfamily	-	21.51% 4e-16 (AAM63125.1)	59.84% 5e-38 (EHI_051440)	OG6_204588
	EIN_081930	408	408	EiMybS8	45-95	Myb-DNA Binding/SANT Superfamily	SANT Superfamily	-	29.63% 5e-14 (CAA0198797.1)	51.88% 1e-33 (EHI_013340)	OG6_204588
	EIN_407300	546	546	EiMybS9	88-138	Myb-DNA Binding/SANT Superfamily	SANT Superfamily	23.64 % 3e-11	19.77% 5e-12 (NP_001330337.1)	46.92% 6e-19 (EHI_038640)	OG6_127050
**Ada2-like**	EIN_359680*	1229	993	EiAda2-Like 1	66-108	ADA2-like ZZ	Histone acetyltransferase complex SAGA/ADA, subunit ADA2 [Chromatin structure and dynamics]	18.75% 3e-13	30.59% 1e-62 (CAD5328117.1)	64.85% 9e-146 (EHI_142140)	OG6_100940
	EIN_390470	1032	1032	EiAda2-like 2	74-116	ADA2-like ZZ	Histone acetyltransferase complex SAGA/ADA, subunit ADA2 [Chromatin structure and dynamics]	20.83% 1e-09	34.38% 2e-60 (CAD5328118.1)	46.86% 9e-92 (EHI_142140)	OG6_100940
**Myb-related**	EIN_020090	423	423	EiMyb-related 1	47-91	Myb-DNA Binding/SANT Superfamily	Myb-DNA Binding/SANT Superfamily	29.1% 4e-17	32% 4e-18 (NP_201038.1)	25.00% 6e-19 (EHI_009930)	OG6_100129
**Zuotin**	EIN_182440*	1658	1596	EiZuotin-like	472-521	DNAJ domain	ZUO1 Superfamily / SANT Superfamily	16.26% 2e-21	30.24% 2e-29 (AAG51437.1)	52.35% 8e-62 (EHI_128200)	OG6_102149
**Bdp1-like**	EIN_223710	366	366	EiBdp1-like 1	45-111	Transcription factor TFIIIB component B', Myb domain	SANT/Myb-like DNA-binding domain-containing protein	- 28.95% 0.017	31.58% 8e-05 (CAB43631.1)	34.29% 2e-10 (EHI_074810)	OG6_105326

								TFIIIB			
	EIN_034860	363	363	EiBdp1-like 2	48-110	Transcription factor TFIIIB component B", Myb domain	SANT/Myb-like DNA-binding domain-containing protein	-<br>30.88%<br>5e-20<br>TFIIIB	25.69%<br>4e-20<br>(CAD5330371.1)	ND	OG6_105326
	EIN_314460	522	522	EiBdp1-like 3	84-126	Transcription factor TFIIIB component B", Myb domain	SANT/Myb-like DNA-binding domain-containing protein	17.65%<br>6e-15	23.70%<br>5e-20<br>(CAD5330371.1)	65.68%<br>3e-37<br>(EHI_009820)	OG6_501849
	EIN_096130	312	312	EiBdp1-like 4	30-90	Transcription factor TFIIIB component B", Myb domain	BDP1 super family	-	30.77%<br>3e-06<br>(CAB43631.1)	40.59%<br>3e-12<br>(EHI_009820)	OG6_501849
	EIN_059360	474	474	EiBdp1-like 5	74-151	Transcription factor TFIIIB component B", Myb domain	SANT/Myb domain	-	25%<br>3e-24<br>(NP_001190961.1)	30%<br>5e-19<br>(EHI_009820)	OG6_501849
						**2R-MYBS**					
I	EIN_284910	519	519	EiMyb1	28-121	Myb-DNA Binding/SANT Superfamily	Transcription repressor MYB5; Provisional	33.63%<br>6e-44	34.35%<br>1e-42<br>(NP_001330339.1)	42.55%<br>2e-43<br>(EHI_063550)	OG6_100129
	EIN_178740	531	531	EiMyb2	26-119	Myb-DNA Binding/SANT Superfamily	Transcription repressor MYB5; Provisional	30.89%<br>4e-46	33.33%<br>1e-43<br>(NP_190575.1)	42.25%<br>1e-39<br>(EHI_063550)	OG6_100129
-	EIN_047330	474	474	EiMyb3	18-112	Myb-DNA Binding/SANT Superfamily	SANT DNA binding domain / Transcription repressor MYB5; Provisional	38.39%<br>2e-40	31.45%<br>2e-42<br>(NP_190575.1)	35.26%<br>1e-41<br>(EHI_063550)	OG6_100129
	EIN_206260	450	450	EiMyb4	16-110	Myb-DNA Binding/SANT Superfamily	PLN03091 super family hypothetical protein; Provisional	34.17%<br>6e-45	35.42%<br>1e-45<br>(NP_190575.1)	34.09%<br>6e-41<br>(EHI_063550)	OG6_100129

	EIN_169560	438	438	EiMyb5	14-109	Myb-DNA Binding/SANT Superfamily	REB1 superfamily	25.19% 1e-44	37.93% 5e-43 (NP_190575.1)	37.40% 1e-37 (EHI_063550)	OG6_100129
	EIN_168610	447	447	EiMyb6	16-110	Myb-DNA Binding/SANT Superfamily	PLN03091 super family hypothetical protein; Provisional	27.83% 1e-42	37.93% 5e-43 (NP_190575.1)	39.69% 1e-37 (EHI_098070)	OG6_100129
	EIN_207200	447	447	EiMyb7	17-79	Myb-DNA Binding/SANT Superfamily	REB1 superfamily	29.29% 4e-45	36.36% 1e-44 (NP_190575.1)	42.34% 5e-41 (EHI_098070)	OG6_100129
	EIN_022390	495	495	EiMyb8	29-122	Myb-DNA Binding/SANT Superfamily	REB1 superfamily	31.03% 5e-46	30.71% 4e-43 (NP_190575.1)	42.31% 2e-39 (EHI_063550)	OG6_100129
	EIN_080130	504	504	EiMyb9	23-114	Myb-DNA Binding/SANT Superfamily	REB1 superfamily	32.65% 4e-46	33.10% 1e-41 (NP_190575.1)	38.40% 2e-40 (EHI_063550)	OG6_100129
II	EIN_276810*	754	702	EiMyb10	31-124	Myb-DNA Binding/SANT Superfamily	Transcription repressor MYB5; Provisional	38.53% 4e-42	33.56% 6e-42 (NP_190575.1)	31.48% 2e-38 (EHI_063550)	OG6_100129
	EIN_307410	507	507	EiMyb11	20-113	Myb-DNA Binding/SANT Superfamily	Transcription repressor MYB5; Provisional	30.58% 5e-46	31.72 7e-46 (VYS56784.1)	47.47% 4e-39 (EHI_063550)	OG6_100129
	EIN_307180	468	468	EiMyb12	19-113	Myb-DNA Binding/SANT Superfamily	Transcription repressor MYB5; Provisional	38.61% 5e-43	32.88% 5e-44 (NP_190575.1)	40.27% 2e-41 (EHI_063550)	OG6_100129
III	EIN_308550	468	468	EiMyb13	19-112	Myb-DNA Binding/SANT Superfamily	Transcription repressor MYB5; Provisional	38.53% 1e-43	34.04% 4e-43 (NP_190575.1)	40.29% 1e-40 (EHI_063550)	OG6_100129
-	EIN_095310	474	474	EiMyb14	30-124	Myb-DNA Binding/SANT Superfamily	PLN03091 super family hypothetical protein; Provisional	37.07% 3e-40	33.87% 1e-40 (NP_195443.1)	51.52% 2e-40 (EHI_153350	OG6_100129

IV	EIN_399710*	1119	918	EiMyb15	161-255	Myb-DNA Binding/SANT Superfamily	REB1 superfamily	35.66% 2e-43	34.27% 2e-42 (NP_195443.1)	68.29% 1e-48 (EHI_098070)	OG6_100129
	EIN_490880	477	477	EiMyb16	41-140	Myb-DNA Binding/SANT Superfamily	REB1 superfamily	38.89% 2e-41	35.83% 1e-42 (NP_195443.1)	39.09% 3e-39 (EHI_063550)	OG6_100129
	EIN_310240	444	444	EiMyb17	31-125	Myb-DNA Binding/SANT Superfamily	REB1 superfamily	45.05% 4e-44	36.36% 3e-44 (NP_195443.1)	44.25% 9e-40 (EHI_098070)	OG6_100129
	EIN_425380	486	486	EiMyb18	32-124	Myb-DNA Binding/SANT Superfamily	Transcription repressor MYB5; Provisional	28.93% 1e-43	33.05% 6e-42 (OAO92063.1)	44.14% 4e-35 (EHI_098070)	OG6_100129
	EIN_046410	504	504	EiMyb19	39-132	Myb-DNA Binding/SANT Superfamily	REB1 superfamily	34.58% 2e-43	34.91% 1e-41 (OAO92063.1)	51.25% 3e-47 (EHI_063550)	OG6_100129
V	EIN_183110	456	456	EiMyb20	31-124	Myb-DNA Binding/SANT Superfamily	PLN03091 super family hypothetical protein; Provisional	33.07% 4e-46	37.01% 3e-46 (VYS56784.1)	38.84% 1e-38 (EHI_098070)	OG6_106827
	EIN_183730	459	459	EiMyb21	31-124	Myb-DNA Binding/SANT Superfamily	PLN03091 super family hypothetical protein; Provisional	38.10% 2e-42	33.08% 2e-42 (CAA0401764.1)	64.10% 2e-39 (EHI_130060)	OG6_106827
	EIN_169190	453	453	EiMyb22	15-108	Myb-DNA Binding/SANT Superfamily	Transcription repressor MYB5; Provisional	29.82% 2e-41	35.77% 2e-41 (AAS58517.1)	46.48% 7e-39 (EHI_168310)	OG6_100129
	EIN_359630	453	453	EiMyb23	29-122	Myb-DNA Binding/SANT Superfamily	PLN03091 super family hypothetical protein; Provisional	35.45% 4e-43	39.05% 2e-42 (CAA0383923.1)	57.58% 4e-44 (EHI_129790)	OG6_106827
	EIN_379820	453	453	EiMyb24	29-122	Myb-DNA Binding/SANT Superfamily	PLN03091 super family hypothetical protein; Provisional	34.43% 5e-44	32.41% 1e-43 (VYS56784.1)	53.62% 5e-42 (EHI_129790)	OG6_106827

-	EIN_168860	495	495	EiMyb25	29-126	Myb-DNA Binding/SANT Superfamily	PLN03091 super family hypothetical protein; Provisional	29.13%<br>9e-46	33.93%<br>3e-42<br>(CAD5329766.1)	46.23%<br>1e-32<br>(EHI_092700)	OG6_106827
-	EIN_405040	615	615	EiMyb26	65-458	Myb-DNA Binding/SANT Superfamily	REB1 superfamily	28.23%<br>4e-40	30.86%<br>1e-43<br>(AAS58517.1)	43.37%<br>5e-36<br>(EHI_053000)	OG6_100129
-	EIN_248780	714	714	EiCDC5-like	10-102	CDC5L_II		22.78%<br>5e-39<br>58.71%<br>8e-41<br>(CDC5)	53.45%<br>2e-55<br>(OAP18307.1<br>CDC5)	78.48%<br>2e-22<br>(EHI_000550)	OG6_102607
**4R-MYBS**											
**SNAP-like**	EIN_267690	1992	1992	EiSNAP-like I	R1 436-599	SANT/Myb domain	SANT/Myb domain	28.22%<br>2e-11<br>39.08%<br>7e-08<br>(SNAP190)	31.22%<br>3e-18	29.97%<br>1e-185<br>(EHI_130710)	OG6_501849

* Intron containing MYB-DBDs encoded genes; - No identity found against *H. sapiens* c-Myb; ND No identity found against *E. histolytica* genome.

[a] Accession number according to NCBI (https://www.ncbi.nlm.nih.gov/) [b] According to OrthoMCL DB.
